# The Association Between Cognitive Status and Cerebral Blood Flow Differs Depending on Reference Region Selection

**DOI:** 10.1002/alz70856_106938

**Published:** 2026-01-08

**Authors:** Krish U Shah, W Hudson Robb, Sarah E Goodale, Dandan Liu, Yukti Vyas, Niranjana Shashikumar, Kimberly R. Pechman, Timothy J. Hohman, Angela L. Jefferson

**Affiliations:** ^1^ Vanderbilt Memory and Alzheimer's Center, Vanderbilt University School of Medicine, Nashville, TN, USA; ^2^ Vanderbilt Memory and Alzheimer's Center, Vanderbilt University School of Medicine, Nashville, TN, USA; ^3^ Department of Biostatistics, Vanderbilt University Medical Center, Nashville, TN, USA; ^4^ Department of Neurology, Vanderbilt University Medical Center, Nashville, TN, USA; ^5^ Department of Medicine, Vanderbilt University Medical Center, Nashville, TN, USA

## Abstract

**Background:**

Studies examining differences in cerebral blood flow (CBF) across the cognitive aging spectrum [cognitively unimpaired (CU), mild cognitive impairment (MCI), dementia] often normalize or adjust regional CBF values using a reference region. Commonly used reference regions include CBF in the putamen or precentral gyrus, though it remains unclear whether normalization is necessary or how reference region choice affects findings. We investigated whether associations between cognitive status and CBF vary by reference region use.

**Methods:**

Vanderbilt Memory and Aging Project participants (*n* = 441, 74% CU, 19% MCI, 7% dementia, 72±10 years old, 49% female) underwent 3T magnetic resonance imaging and neuropsychological evaluation. Pseudo‐continuous arterial spin labeling assessed CBF in total cerebral grey matter and cortical lobar gray matter (frontal, parietal, temporal, occipital) regions of interest (ROI) and in reference regions (putamen, precentral gyrus). Ordinary least squares regressions related cognitive status to CBF in each ROI, adjusting for age, sex, race/ethnicity, education, Framingham Stroke Risk Profile, *apolipoprotein E‐*ε4 status, and, when appropriate, reference region CBF. ANOVA and pairwise comparisons (MCI–CU, dementia–MCI) followed.

**Results:**

Without reference region adjustment, CBF differed by cognitive status (*p*‐values<0.02) in all ROIs, though CU and MCI did not differ in pairwise comparisons (*p*‐values>0.10). Relative to individuals with MCI, individuals with dementia had significantly lower CBF in total, frontal, temporal, and, particularly, parietal (β=‐7.9 mL/100g/min, *p* = 0.005) gray matter. Compared to adjusting for precentral gyrus CBF, adjusting for putamen CBF resulted in more associations between cognitive status and CBF. After adjusting for putamen CBF, CBF was lower in MCI relative to CU across all ROIs (*p*‐values<0.01), but differences between MCI and dementia were attenuated compared to models without a reference region (e.g., parietal lobe β=‐4.8 mL/100g/min, *p* = 0.01).

**Conclusions:**

Adjusting for CBF in a reference region, particularly the putamen, may enhance the detectability of CBF differences early along the cognitive aging spectrum. However, including a reference region may reduce sensitivity to detect CBF differences among individuals already exhibiting cognitive impairment, potentially because reference regions become hypoperfused. Future research should determine whether adjusting for CBF in a reference region enhances the detection of AD biomarker positivity.

